# CIG-DB: the database for human or mouse immunoglobulin and T cell receptor genes available for cancer studies

**DOI:** 10.1186/1471-2105-11-398

**Published:** 2010-07-27

**Authors:** Yoji Nakamura, Tomoyoshi Komiyama, Motoki Furue, Takashi Gojobori, Yasuto Akiyama

**Affiliations:** 1 Immunotherapy Division, Shizuoka Cancer Center Research Institute, 1007 Shimonagakubo, Nagaizumi-cho, Sunto-gun, Shizuoka, 411-8777, Japan; 2 Department of Clinical Pharmacology, Tokai University School of Medicine, 143 Shimokasuya, Isehara, Kanagawa, 259-1193, Japan; 3 Bioinformatics Institute for Global Good Inc., Kitashinagawa 3-6-9, Shinagawa-ku, Tokyo, 140-0001, Japan; 4 Center for Information Biology and DNA Data Bank of Japan, National Institute of Genetics, Yata 1111, Mishima, Shizuoka, 411-8540, Japan; 5 Current Address: National Research Institute of Fisheries Science, Fisheries Research Agency, 2-12-4 Fukuura, Kanazawa, Yokohama, Kanagawa, 236-8648, Japan

## Abstract

**Background:**

Immunoglobulin (IG or antibody) and the T-cell receptor (TR) are pivotal proteins in the immune system of higher organisms. In cancer immunotherapy, the immune responses mediated by tumor-epitope-binding IG or TR play important roles in anticancer effects. Although there are public databases specific for immunological genes, their contents have not been associated with clinical studies. Therefore, we developed an integrated database of IG/TR data reported in cancer studies (the Cancer-related Immunological Gene Database [CIG-DB]).

**Description:**

This database is designed as a platform to explore public human and murine IG/TR genes sequenced in cancer studies. A total of 38,308 annotation entries for IG/TR proteins were collected from GenBank/DDBJ/EMBL and the Protein Data Bank, and 2,740 non-redundant corresponding MEDLINE references were appended. Next, we filtered the MEDLINE texts by MeSH terms, titles, and abstracts containing keywords related to cancer. After we performed a manual check, we classified the protein entries into two groups: 611 on cancer therapy (Group I) and 1,470 on hematological tumors (Group II). Thus, a total of 2,081 cancer-related IG and TR entries were tabularized. To effectively classify future entries, we developed a computational method based on text mining and canonical discriminant analysis by parsing MeSH/title/abstract words. We performed a leave-one-out cross validation for the method, which showed high accuracy rates: 94.6% for IG references and 94.7% for TR references. We also collected 920 epitope sequences bound with IG/TR. The CIG-DB is equipped with search engines for amino acid sequences and MEDLINE references, sequence analysis tools, and a 3D viewer. This database is accessible without charge or registration at http://www.scchr-cigdb.jp/, and the search results are freely downloadable.

**Conclusions:**

The CIG-DB serves as a bridge between immunological gene data and cancer studies, presenting annotation on IG, TR, and their epitopes. This database contains IG and TR data classified into two cancer-related groups and is able to automatically classify accumulating entries into these groups. The entries in Group I are particularly crucial for cancer immunotherapy, providing supportive information for genetic engineering of novel antibody medicines, tumor-specific TR, and peptide vaccines.

## Background

The immune system is inherent in vertebrates and provides protection against toxic substances or infectious diseases. Two antigen receptor proteins, immunoglobulin (IG) expressed on B lymphocytes or secreted by plasma cells, and the T-cell receptor (TR), expressed on T lymphocytes, are key molecules for humoral immunity and cell-mediated immunity, respectively [1]. Each of these proteins consists of two chain types, called light and heavy chains for IG (there are two identical light chains and two identical heavy chains in an IG), and alpha and beta chains, or gamma and delta chains for TR. Each chain contains, at its N-terminal end, a variable (V) domain which participates in antigen recognition. The V domain is encoded by two or three genes, a V gene, a diversity (D) gene (for heavy, beta and delta chains) and a joining (J) gene, which rearrange through somatic recombination [2]. In the V domain, three complementarity determining regions (CDRs), which are especially sequence-diversified, contact antigenic epitopes. In particular, the third CDR (CDR3) is the most diversified among the CDRs at the junction of V(D)J recombination and is considered crucial for the recognition of epitopes [3-5].

Cancer cells proliferate abnormally compared to normal cells, often expressing proteins (tumor-associated antigens) that cannot be seen in normal developmental stages [6]. In cancer studies, monitoring the immune status of patients is thus very important for diagnosis, as expression of an autoantibody [7] and the activation of cytotoxic T lymphocytes (CTLs) [8] specific to tumor-associated antigens are observed. In hematological tumors, such as leukemia or lymphoma, IG and TR themselves are the subject of investigation, because the encoding genes are often mutated by translocation in tumor B or T cells [9]. Moreover, in recent years, these antigen receptor proteins have attracted attention in the field of cancer immunotherapy to elevate the patient's immune response against tumor cells with few side-effects [10,11]. In cellular immunotherapy, T cells recognizing tumor-associated antigens can be administrated back to patients after *ex vivo *culture and processing for immune response enhancement.

During the last decade, monoclonal antibodies have been sought and engineered as candidates for molecular target drugs [12]. These molecules can recognize the cancer cells expressing tumor-associated antigens with high affinity and selectivity, triggering anticancer effects [12,13], such as complement dependent cytotoxicity, antibody-dependent cellular cytotoxicity, inhibition of angiogenesis, and induction of apoptosis. In general, the source of antibody medicines is the human or mouse: (i) fully murine, (ii) chimeric with V domains from the mouse and constant regions from the human, and (iii) humanized or human antibodies have been developed [12]. For instance, trastuzumab (trade name Herceptin), a humanized antibody that targets the human epidermal growth factor receptor type 2 protein, has shown success in the treatment of breast cancer [14]. Around 10 antibody medicines in cancer therapy are now approved and another 30 are being assessed in clinical trials in the USA [12].

Cancer vaccines are another type of immunotherapy, in which partial epitope peptides of tumor-associated antigens are administered to patients to potentiate CTL activity [15]. TR on CD8+ T cells recognize the peptide vaccines bound with human leukocyte antigens (HLA), which are displayed by antigen-presenting cells, and enhance cytotoxic activity against cancer cells carrying the peptides [16]. Each peptide vaccine is, in general, 9-10 amino acids long and selected according to the patient's HLA allele type.

These immunotherapeutic studies have emphasized the importance of genetic engineering of IG and TR proteins and peptide vaccines at the sequence level [17-21]. Currently, there are several immunological gene databases available online, such as IMGT^®^, the international ImMunoGeneTics information system^® ^[22] and the Immune Epitope Database and Analysis Resource (IEDB) [23]. Although the contents of these databases are well-annotated and specific to genetic information on IG, TR, and their epitopes, there are still gaps between this information and the clinical application in cancer research. In particular, the information supplied is too broad for clinicians and pharmacologists (for example, such databases store the data from a large variety of organisms and the majority are unrelated to cancer) to easily obtain the information required for patient-specific treatment. To address these issues, we have developed a freely accessible database, the Cancer-related Immunological Gene Database (CIG-DB). The database integrates the information on IG, TR, and epitopes reported in cancer studies, and presents sequence analysis tools, and structural data.

## Construction and content

### Data integration

The CIG-DB is a semi-automated database consisting of four tables, two of which are for IG and TR proteins, respectively. All the included proteins are only derived from the human or mouse. The other two tables are for epitopes of IG or TR, where we collected the amino acid sequences from a variety of organisms regardless of whether they were cancer-related or not. Since the available amount of cancer-related epitope data is still small, a large number of sequences are considered useful for further comparative analysis.

First, to thoroughly check all public IG/TR proteins of human and mouse origins, we downloaded their annotation data from GenBank/DDBJ/EMBL by keyword search. Then, the Geninfo Identifier (GI) and accession numbers, full amino acid sequences, and references were extracted and tabularized as a local proto-database. CDR3 sequences were also extracted from the full sequences using a pattern-matching program to find the N- and C-terminal borders. The program detects conserved motifs flanking the CDR3 region using regular expressions, such as the second cysteine and its adjacent residues as the N-flanking sequence and a WGXG motif as the C-flanking sequence. The V and J gene repertoires were assigned according to IMGT^® ^nomenclature [24,25] and BLAST [26] matching. Furthermore, we obtained structural data of the IG/TR proteins from the Protein Data Bank (PDB) [27], if any, and the information was merged with the GenBank annotation. We thus obtained a total of 32,240 entries for IG and 6,068 for TR, as of October 1, 2009 (Table 1). Next, for each of the entries, we obtained the corresponding 2,740 MEDLINE reference data from PubMed at the National Center for Biotechnology Information (Table 2). The number of references is approximately one order of magnitude smaller than that of the receptor sequences, because multiple IG or TR sequences are often reported in a single reference. We then selected 578 MEDLINE reference texts in which cancer-related keywords ("melanoma," "carcinoma," "sarcoma," etc.) are contained in at least one of either the MeSH terms, title, or abstract sentences. When we annotated the IG/TR entries according to the selected references, we noticed that the IG/TR entries screened were used for the following cases in cancer studies: (i) studied in the context of cancer therapy (specific TR and antibodies monitored after administration of peptide vaccines in immunotherapy, monoclonal antibodies specific to cancer markers, etc.) and (ii) isolated from hematological tumors such as leukemia, lymphoma, and myeloma (survey of mutation in IG or TR gene locus, etc.). Therefore, we manually classified the IG/TR entries into these two groups, named Groups I (cancer therapy) and II (hematological tumors), and the corresponding entries were tabularized in the same format as the above-mentioned proto-database. Finally, we obtained a total of 2,081 cancer-related receptor entries, 1,605 for IG and 476 for TR (Table 1).

**Table 1:** CIG-DB statistics as of 1 October 2009

Data content	IG	TR	Total
Screened from NCBI and PDB	32240	6068	38308
Cancer-related	1605	476	2081
			
Human	879	318	1197
Mouse	726	158	884
			
Group I^a^	397	214	611
Group II^b^	1208	262	1470
			
Chains			
Light	791		
Heavy	814		
Alpha		185	
Beta		288	
Gamma^c^		3	

Epitope sqeuences	772	148	920

^a^This group is involved in cancer therapy such as antibody medicines.

^b^This group is involved in expression in hematological tumors.

^c^A minor TCR allele.

**Table 2:** Classification of cancer-related references in CIG-DB

Data content	IG	TR	Total
Collected from PubMed	2054	686	2740
			
Screened by keywords	446	132	578
			
Manually classified^a^			
Group I	120	34	
Group II	139	37	
(Group III)	187	61	

^a^Groups I and II are the same as in Table 1. Group III is a group of wrongly screened references.

Regarding epitopes, the interaction between IG/TR and an epitope is the specific focus of this database. The source of epitope sequences was public databases: the IEDB, Bcipep [28], and the HIV sequence database [29], and where possible the PDB, if epitopes were crystallized as bound with IG or TR. We then extracted the protein or peptide epitopes and selected those whose antigen receptor sequences are known. The matching criteria were as follows: (i) the complex structure of the antigen receptor and epitope are already in the PDB, (ii) for the epitopes from the IEDB, the GI numbers of the receptors are found in the IG/TR tables of the CIG-DB, and (iii) manual check of references. As a whole, we obtained a total of 920 epitope sequences, 772 for IG, and 148 for TR (Table 1). Antigen-presenting cells display as T cell epitopes around 9-mer peptides, and TR epitopes are therefore always of linear sequences. In the case of IG epitopes, conformational ones neighbored through antigen folding are possible. To show the key residues involved in the interaction with the antigen receptor, residues were highlighted based on the distances of the residues to the receptor's CDR3 (< 4 angstroms).

### Reference clustering and classification

**Figure 1:**
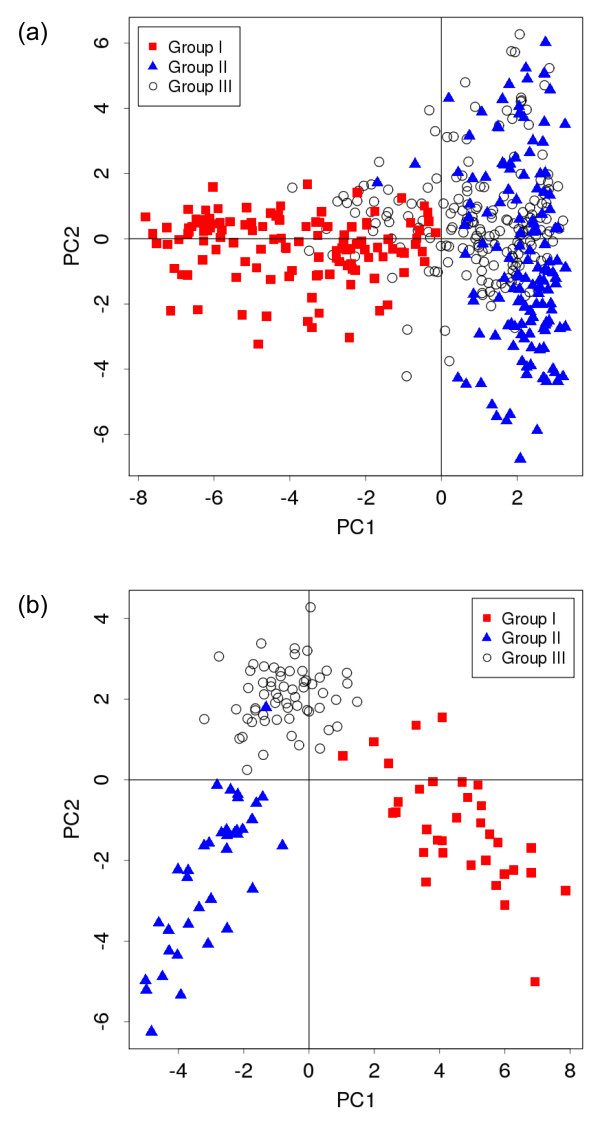
**The result of principal component analysis on cancer-related immunoglobulin (IG) and T-cell receptor (TR) references**. PC1 and PC2 indicate scores of the first and second principal components, respectively. (a) IG references and (b) TR references.

In the current version of the CIG-DB, as mentioned above, we manually checked the references to classify the IG and TR entries into two cancer-related groups. We further prepared an automated classification method based on text mining, training, and clustering algorithms. For training, we appended one additional group (Group III) of unrelated references (Table 2), which was wrongly selected by cancer-related keywords and dropped by manual classification. First, we parsed each of the MEDLINE text format data of the three groups using the *tm *library of the R program and computed a term frequency vector for the words occurring in the MeSH terms, title, and abstract, after processing and stemming. Figure 1 shows the result of principal component analysis (PCA), which plots the first and second principal components (PC1 and PC2) using all the term frequency vectors, suggesting that the three groups are distributed separately from each other for both IG and TR references. Next, we averaged and merged all the frequency vectors of the references in each group and calculated a canonical discriminant function, using the Mahalanobis distance between any of the references and each of the three groups, as shown below:

Here, *d*(*x*_*i*_, μ_*j*_) is the distance between a term frequency vector (*x*_*i*_) of reference *i *and the mean vector (μ_*j*_) of group *j *(*j = 1,2,3*), Σ_*j*_^-1 ^is an inverse matrix of the variance-covariance matrix of group *j*, and *T *indicates the transposition of the vector. In the discriminant function, each reference was classified into the group in which *d *became a minimum value. To validate the discriminant function, we performed a leave-one-out cross validation (Table 3). The accuracy rates of classification were very high: 94.6% for IG and 94.7% for TR.

**Table 3:** Validation of reference classification by canonical discriminant analysis

		Predicted groups				
				
Protein and group^a^		Group I	Group II	Group III	Total	Accuracy (%)
IG	Group I	**111**	0	9	120	92.5
	Group II	0	**128**	11	139	92.1
	Group III	0	4	**183**	187	97.9
	Total				**422/446**	**94.6**

TR	Group I	**31**	0	3	34	91.2
	Group II	0	**33**	4	37	89.2
	Group III	0	0	**61**	61	100.0
	Total				**125/132**	**94.7**

^a^Groups I, II and III are the same as in Tables 1 and 2.

## Utility

### Reference and sequence search

The CIG-DB provides two search engines: (i) sequence search and (ii) reference search, which users can select on the home page.

#### Sequence search

Users can perform keyword searches by GI numbers, amino acid sequences, or GenBank accessions of IG/TR/epitope entries. Users can select eight search conditions, "Contains/Does not contain," "Equals/Does not equal," "Starts with/Does not start with," and "Ends with/Does not end with." The search result is shown as a table that can be sorted in ascending or descending order. Most importantly, the sequence search for IG or TR entries can be focused on cancer-related sequences by checking the filter option box. In a resultant table, GI numbers and references are linked to GenBank and PubMed online, respectively, and the reference IDs (i.e., PubMed IDs) shown are numbered as "1" (cancer therapy) or "2" (hematological tumors). The same search engine is also available for epitope entries.

#### Reference search

**Figure 2:**
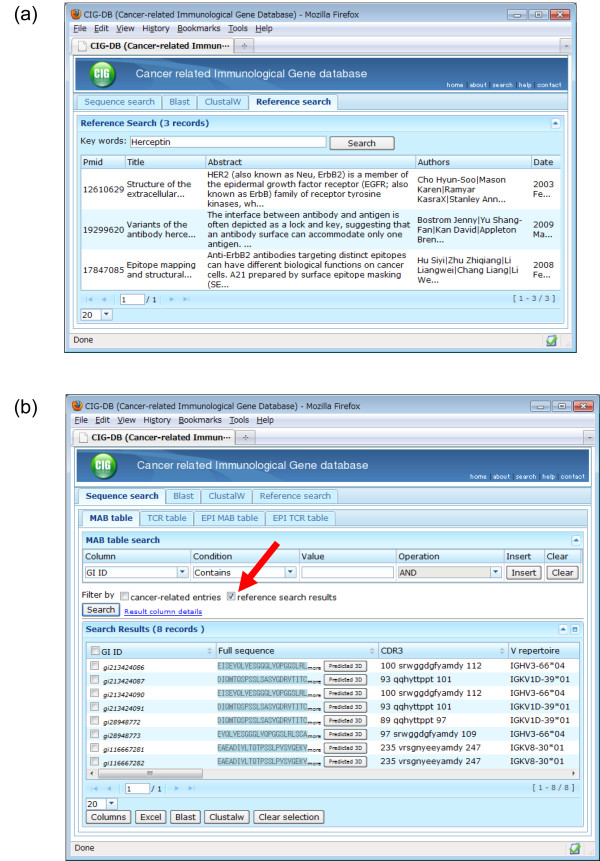
**A search interface of the Cancer-related Immunological Gene Database (CIG-DB)**. (a) An example of search results where the word "Herceptin" is queried against references in the CIG-DB. (b) The subsequent sequence search result from (a). A check box for reflecting the reference search result to sequence search is indicated by a red arrow.

Users also can search for IG/TR entries by MeSH terms, words in the title or abstract, names of authors, journals, or publication dates, and thus narrow down research fields of interest. This feature is an advantage of the CIG-DB, because such a detailed reference search is not offered by other public immunological databases. Figure 2a shows an example of a search result using the name of an antibody medicine against breast cancer, "Herceptin." The reference search result can be reflected to the subsequent sequence search to corresponding IG/TR/epitope entries by a check box, so that users can see amino acid sequences that were reported in the matched reference (Figure 2b).

### Sequence analysis tools

**Figure 3:**
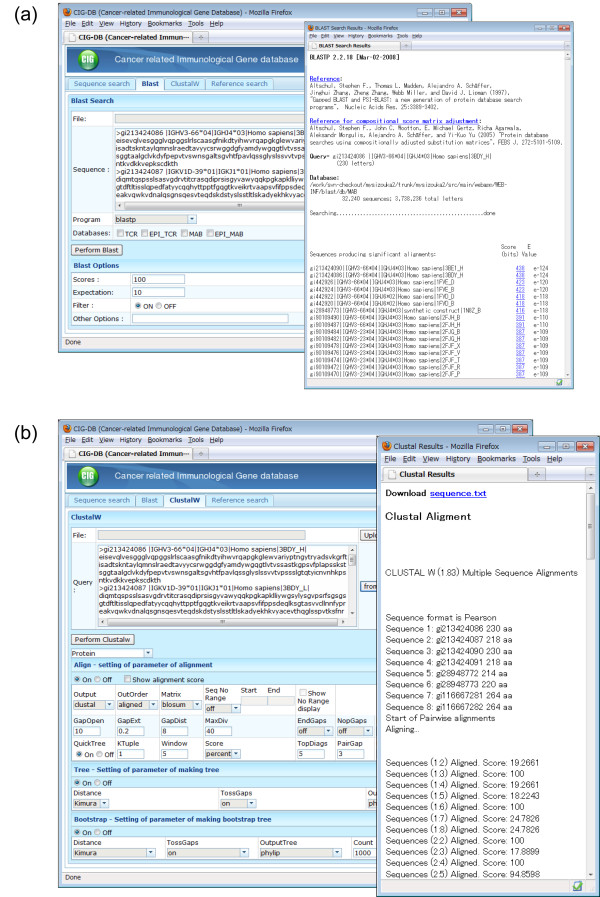
**Sequence analysis tools of CIG-DB**. (a) An example of BLAST query and result pages. (b) An example of CLUSTALW query and result pages. Both analyses were performed from the search result page in Figure 2b.

In clinical study, one may determine the sequences of IG or TR specific to tumor-associated antigens from a patient's B or T cells and compare these with public sequences. The CIG-DB thus provides BLAST (Ver. 2.2) and CLUSTALW [30] (Ver. 1.83) tools for sequence similarity search and alignment (Figure 3). Here users can compare the in-house sequences with cancer-related sequences in the CIG-DB, or with all public IG/TR sequences regardless of cancer studies in the proto-database. It is also possible to perform the analysis using only the sequences within the CIG-DB. For example, after a reference or sequence search, the result can be subsequently utilized for such comparative analyses. The matched sequence list by BLAST and multiple alignment by CLUSTALW are downloadable in text format.

### 3D structure visualization and modeling

**Figure 4:**
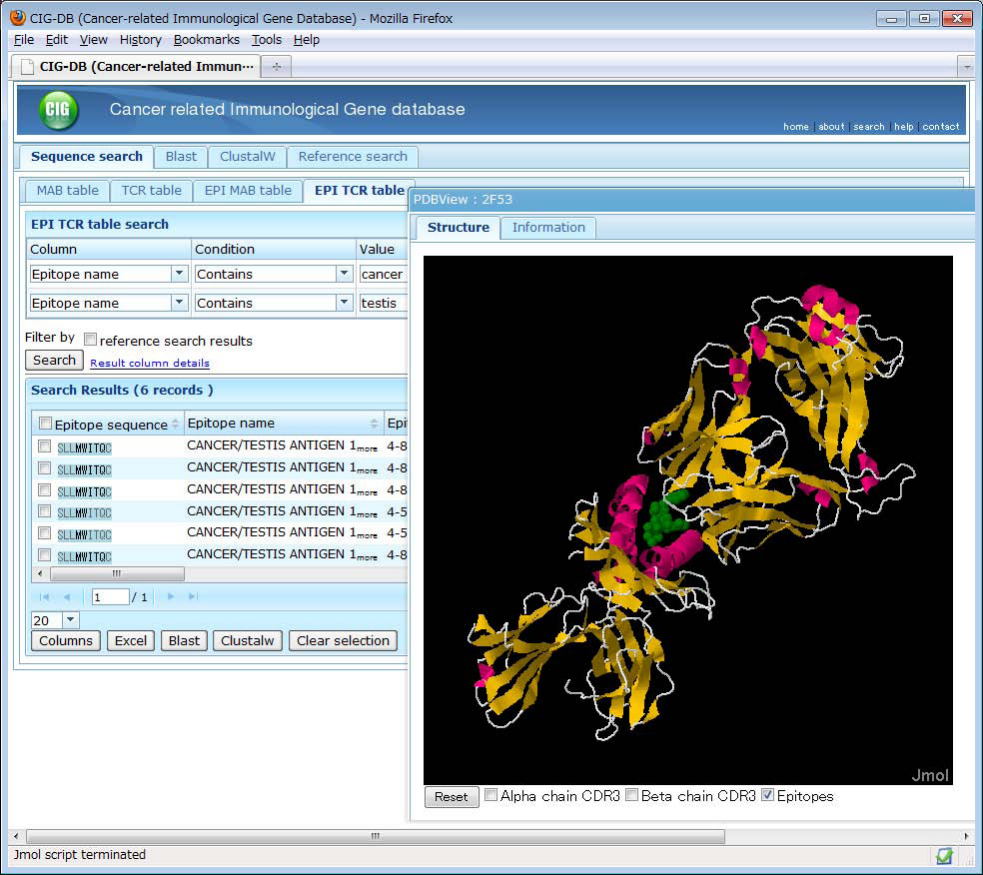
**A 3D viewer of CIG-DB**. The entries containing two words, "cancer" and "testis," were searched against the column of "Epitope name" in TR-epitope table (EPI TCR table). A 3D structure of the top hit (PDB code = 2F53, the complex between the T-cell receptor and cancer/testis antigen 1B peptide) is shown. The epitope molecule is highlighted in green by checking a box in the viewer.

For the entries merged from the PDB, which are either for a receptor alone or a receptor-epitope complex, the tertiary structures are visualized by a JAVA applet based on Jmol [31] (Figure 4). This viewer can be customized so that interacting residues are highlighted. Particularly, in epitope tables, the epitope structure derived from the PDB is viewed as bound with the antigen receptor, and the applet allows users to investigate the interaction between CDR3 and epitope residues (atomic distance, etc.). In addition, the CIG-DB offers predicted structures for non-PDB IG/TR entries using MODELLER [32], a protein 3D modeling software based on the homology modeling method. In MODELLER, an amino acid sequence is compared against a 1D sequence database consisting of known PDB structures. PDB structures whose sequences are similar to that of the query are used as templates in 3D alignment, followed by energy optimization of aligned structures. In the CIG-DB, the templates are local data of human and mouse IG/TR proteins collected from the PDB, whose backbone structures are sufficiently conserved. For each of the non-PDB IG/TR entries, five predicted structures and their scores (objective functions) in optimization are calculated, and users can download these models in PDB format for further refinement by structure calculation (e.g., molecular dynamics or docking simulation).

### Database implementation and update

The CIG-DB is a Java web application developed using an open source Ajax framework, ZK, which uses MySQL as a database backend. As a high performance search engine, this database is equipped with Apache Lucene. The application runs on a servlet container, Apache Tomcat. An update of the basic contents is semi-automatic, achieved by Perl and Shell scripts. In the future, the reference classification script by R programs will be integrated into the automation.

## Discussion

Immunotherapy is now an effective treatment for cancer, where the information on cancer-antigen-specific IG or TR as well as the epitopes, is essential for its use. In particular, genetic engineering, such as the improvement of antibody sequences as molecular target drugs or the design of epitope peptides for cancer vaccines, are of great interest to clinicians and pharmacologists. For such studies, a sequence-based database for cancer immunological genes has potential utility. In addition, sequence comparison tools and structural data are useful for genetic engineering, combined with library screening methods in a laboratory [33,34]. Our database, the CIG-DB, thus may meet the needs of researchers involved in such cancer studies. Moreover, this database is also designed as a reference-based platform, equipped with a search engine by MeSH terms, title, and abstract words (Figure 2a). In previous public databases, one could find the immunological gene sequences, but the references were not always related to cancer, or one could search for cancer-related papers, but the sequences frequently were undetermined. Our database avoids such inconveniences and users can easily obtain the cancer-related IG/TR sequences that are available. For comparison, IMGT also provides with the data sheets on therapeutic monoclonal antibodies related to oncology http://www.imgt.org/mAb-DB/query, but does not yet encompass the information about cancer vaccines and TRs specific to tumor-associated antigens. The IEDB is an exhaustive epitope resource and covers a wide range of experimental details, but the search option is not optimized to find cancer-related protein/peptide sequences.

Considering their application in cancer studies, IG and TR proteins are derived from two mammals, the human and the mouse. In this study, it was found that such cancer-related IG/TR proteins can be classified into two groups, namely, cancer therapy (Group I) and hematological tumors (Group II). One more group (Group III) of unrelated references includes papers mainly involving experiments about hybridoma using "myeloma" or those about irrelevant "tumors," which are therefore wrongly selected by keyword matching. The classification of cancer-related IG and TR is thus very important for ensuring a high quality of the CIG-DB. Maintenance of the current version of the CIG-DB is semi-automated from initially obtaining the sequence data to presenting the final tables on the graphical user interface. Manual operation is only necessary for the very classification of IG and TR annotation entries into the two groups by checking their references, but this method would be a troublesome task for future updates. To reduce this burden, we prepared a computational classification program inside the database. As a basis for this step, PCA results suggest that the two groups can be discriminated from each other by the term frequencies in MEDLINE texts (Figure 1). Although Group III overlaps somewhat with Group II in the case of IG references, the distributions can be statistically discriminated from each other (*P *< 0.001, multivariate analysis of variance). In this study, we calculated a canonical discriminant function using a set of references that were classified manually in the current version of the database as a training dataset. We evaluated the function by leave-one-out cross validation and obtained high accuracy rates (Table 3), strongly suggesting that our method can be applicable for an automated update. For the next update, this procedure may possibly be integrated into the maintenance programs of the CIG-DB. Alternatively, we will perform a manual classification and calculate the discriminant function again over a few more updates, further improving the accuracy of the database.

This database provides structural data of antigen receptors. Particularly, for all non-PDB IG/TR entries, the five predicted structural models are available in PDB format, which allows users to study the interaction between IG/TR and the epitope using molecular dynamics or docking simulation. This is an advantage of our database over other public databases, such as IMGT and the IEDB which provide only known structures. It should be noted that the combinatorial study using these tools and IG/TR data in Group I are efficient for cancer immunotherapy, in which genetic design and engineering are performed for developing novel antibody medicines, tumor-specific TR, and peptide vaccines with potent anticancer effects. Recently, new technologies have allowed rapid cloning of effective antibodies to specific antigens [35,36]. It is thus likely that a large number of antibodies and TR for tumor-associate antigens will be open for genetic improvement in the near future. Since the CIG-DB is a semi-automated database, the latest information will be quickly reflected there. We believe that accumulation of IG/TR/epitope data will enhance the usefulness of this database in clinical cancer studies.

## Conclusions

The CIG-DB is designed to serve as a bridge between immunological gene data and cancer studies, presenting annotations of IG, TR, and their epitopes. In its current version, the database has 2,081 cancer-related human and murine receptor entries (1,605 for IG and 476 for TR), and 920 entries for epitopes bound with receptors from a variety of organisms. Regarding IG and TR proteins, this database further provides a helpful guide to two detailed groups; one for cancer therapy and the other for hematological tumors. For the next update, we have developed a powerful method to automatically classify cancer-related entries into these two groups. The high precision (~95% accuracy) shown in validation assessments is promising for the efficient performance of the database. Moreover, the CIG-DB is equipped with sequence- and reference-search engines and analysis tools, the results of which can be utilized for advanced studies. In particular, the database will play important roles in cancer immunotherapy, by integrating the accumulating patient-specific IG/TR/epitope sequence data by novel cloning technologies.

## Availability and requirements

The CIG-DB is available without charge or registration at http://www.scchr-cigdb.jp/. We have confirmed that the web site can be viewed by Internet Explorer 7 or later, Safari 3, and Firefox 3. The Java Runtime Environment (JRE 1.5 or higher) is required for displaying 3D structures by Jmol applet.

## Authors' contributions

YN designed the database, developed the core scripts and wrote the manuscript. TK and YA conceived the study. MF developed the Web interface. TG participated in coordinating the study and helped to draft the manuscript. All authors read and approved the final manuscript.
